# Facilitators and barriers to cervical cancer screening among women living with HIV: a systematic review of qualitative studies

**DOI:** 10.3389/fpubh.2026.1809112

**Published:** 2026-06-22

**Authors:** Yali Zhou, Xutong Zheng, Dan Shao, Fang Cheng

**Affiliations:** 1 Wuhan Jinyintan Hospital, Tongji Medical College of Huazhong University of Science and Technology, Wuhan, China; 2 Hubei Clinical Research Center for Infectious Diseases, Wuhan, China; 3 Wuhan Research Center for Communicable Disease Diagnosis and Treatment, Chinese Academy of Medical Sciences, Wuhan, China; 4 Joint Laboratory of Infectious Diseases and Health, Wuhan Institute of Virology and Wuhan Jinyintan Hospital, Wuhan, China; 5 Department of Public Service, The First Affiliated Hospital of China Medical University, Shenyang, China

**Keywords:** barriers, cervical cancer, facilitators, HIV, qualitative review, women

## Abstract

**Background:**

Evidence suggests that 58% of new cervical cancer cases worldwide occur among women living with HIV. However, compared with HIV-negative women, this population has significantly poorer access to healthcare services for cervical cancer screening. There is a need to better understand the factors influencing their uptake of cervical cancer screening within the context of their lives. This study aims to objectively evaluate and synthesize qualitative studies on the facilitators and barriers to cervical cancer screening among women living with HIV.

**Methods:**

A systematic search was conducted in four databases—Web of Science, Embase, PubMed, and CINAHL—covering the period from January 2000 to December 2024. The included studies were qualitatively reviewed according to the Enhancing Transparency in Reporting the Synthesis of Qualitative Research (ENTREQ) guidelines. A meta-aggregative approach was used to summarize and categorize the identified facilitators and barriers.

**Results:**

A total of 10 articles were included in the study, identifying 27 facilitators and 79 barriers. These factors were categorized into two main themes and six sub-themes.

**Conclusion:**

Women living with HIV face specific barriers to participating in cervical cancer screening. The findings of this review provide evidence to promote cervical cancer screening in this vulnerable population. Future research should focus on increasing the engagement of women living with HIV in cervical cancer screening to develop new strategies for their long-term participation and to contribute to the elimination of cervical cancer at local and global levels.

**Trial Registration:**

identifier [CRD42024498012].

## 1 Introduction

Cervical cancer (CC) is one of the most common gynecological malignancies among women globally, ranking fourth in terms of incidence and mortality (1). However, cervical cancer is preventable through effective measures, and early diagnosis followed by timely and effective treatment can even lead to complete recovery (2, 3). Studies have shown that nearly all cases of cervical cancer originate from persistent infection with high-risk human papillomavirus (HPV), a common pathogen of genital tract infections primarily transmitted through sexual contact (4). Currently, preventive measures for cervical cancer include HPV vaccination, cervical cancer screening, and treatment of precancerous lesions (5), with an increasing number of countries opting for primary HPV screening (6). Individuals detected with oncogenic HPV in screening tests can be monitored and retested or referred for colposcopy, with treatment for precancerous lesions provided if necessary. Through these screening programs, cervical cancer-related mortality has been significantly reduced, and survival rates have increased by 92% (7). However, there are significant disparities among populations participating in screening programs.

In 2020, the World Health Organization (WHO) released the Global Strategy to Accelerate the Elimination of Cervical Cancer, aiming to reduce new cases by more than 40% and prevent 5 million related deaths by 2050 through key measures such as vaccination, screening, and treatment. The strategy specifically emphasizes the need for comprehensive, evidence-based interventions, including screening and treatment services, for people living with HIV (5). According to 2018 statistics, 58% of new cervical cancer cases among women were attributed to HIV infection (73). Compared with HIV-negative women, this population has significantly poorer access to healthcare services for cervical cancer screening, due to reasons such as delayed clinical examinations (9), lack of screening facilities in healthcare institutions (10), limited service accessibility (including economic and transportation issues) (11), and personal factors, including lack of knowledge about cervical cancer and screening (8), and unfavorable attitudes toward cervical cancer screening (12).

Screening-based strategies have been reported as effective means to improve early diagnosis efficiency. In Asia, several countries, including China, India, Indonesia, Japan, South Korea, and Thailand, have established national cervical cancer screening programs aimed at preventing the occurrence of cervical cancer cases (13). According to a retrospective cohort study in Estonia from 2009 to 2018, women living with HIV had average annual coverage rates for opportunistic and organized screening that were 4.14% and 8.3% lower, respectively, than those of HIV-negative women (14). This finding is consistent with the conclusions of a systematic review on cervical cancer screening among women living with HIV (WLWH) in low- and middle-income countries, indicating that low awareness of cervical cancer and screening knowledge among WLWH is a major reason for low screening rates (15). The lower screening rates among WLWH have led to a detection rate of 5.4% for precancerous lesions and 0.8% for suspected invasive cervical cancer, a problem emphasized in studies, especially given the significantly increased risk of cervical cancer among women living with HIV (WLWH) (16). Therefore, promoting cervical cancer screening among WLWH is of vital importance.

Previous studies have highlighted unique barriers faced by specific populations in participating in cervical cancer screening, including stigma and discrimination associated with HIV infection, which may deter them from seeking screening (17). Additionally, time-role conflicts, concerns about privacy breaches, and potential discomfort during the screening process can all pose significant burdens for WLWH (18). To date, only the study by Birye (19) has quantitatively assessed the uptake rate of cervical cancer screening and its associated factors among women living with HIV. However, that study employed a quantitative meta-analytic approach and did not integrate qualitative research. As a result, it is unable to capture women's subjective experiences, emotional barriers, or sociocultural facilitators. Given the importance of cervical cancer screening for this population, this study aims to deeply integrate the barriers and facilitators influencing screening participation among WLWH, providing necessary information support for cervical cancer prevention and clinical practice, laying the foundation for future research, and developing new strategies to promote long-term participation in cervical cancer screening among WLWH.

### 1.1 Aim

The aim of this study is to critically evaluate and synthesize qualitative research on the attitudes, experiences, and facilitators and barriers to participation in cervical cancer screening among WLWH.

## 2 Methods

### 2.1 Protocol registration and reporting guidelines

This systematic review utilized the Enhancing Transparency in Reporting the Synthesis of Qualitative Research (ENTREQ) checklist to report the process and results of the synthesis (20). It was also registered on the International Prospective Register of Systematic Reviews (PROSPERO) with the registration number CRD42024498012.

### 2.2 Searching strategy

Two researchers developed the search strategy to identify reports on the thematic factors related to cervical cancer screening among WLWH. Four databases were searched: Web of Science, Embase, PubMed, and CINAHL, each with a tailored search strategy. The search terms used in the databases included: “HIV,” “Human immunodeficiency,” “Acquired immunodeficiency syndrome,” “Acquired immune deficiency syndrome,” “AIDS,” “female,” “woman,” “women,” “Cervical screening,” “pap,” “Papanicolaou test,” “cervical smear,” “early detection of cancer,” “neoplas,” and “human papilloma virus.” The results were limited to journal articles or theses published in English before December 2024. Conference abstracts, research protocols, and social commentaries were manually excluded. Additionally, we hand-searched the reference lists of target articles to identify eligible studies. The search strategies used are detailed in Supplementary File 2.

### 2.3 Eligibility criteria

Studies were included if they met all the following criteria: (1) Utilized a qualitative research design (e.g., phenomenology, grounded theory, case study, ethnography, action research, or other qualitative methods); (2) Recruited WLWH aged 18 years or older; (3) Investigated barriers and facilitators to cervical cancer screening. In this review, barriers were defined as obstacles or negative feedback encountered by participants, as well as challenges they faced in undergoing or participating in screening. Facilitators were described as factors or positive feedback that attracted participants to cervical cancer screening; (4) Were peer-reviewed articles or theses published in English. Studies were excluded if they met any of the following criteria: (1) Used a mixed-methods design with inseparable qualitative data; (2) Full-text articles were unavailable; (3) Included WLWH with a history of total or partial hysterectomy, as these individuals no longer required cervical cancer screening.

Mixed-methods studies were included only when qualitative data could be clearly extracted. Studies in which qualitative findings could not be disaggregated from quantitative results were excluded.

### 2.4 Study selection

Study selection followed the guidelines for systematic reviews (21). The initial search results from the databases were imported into Endnote 21 for screening. After removing duplicates, the titles and abstracts were reviewed to exclude reviews, quantitative studies, and articles unrelated to the topic. Finally, the most eligible studies were included after reading the full texts. At each screening stage, at least two trained reviewers (ZYL, ZXT, SD) independently assessed the articles. Any disagreements were resolved through discussion to reach a consensus.

### 2.5 Quality appraisal of eligible studies

Two independent researchers (ZYL & ZXT) rigorously evaluated the methodological quality of the included studies using the Checklist for Qualitative Research (Critical Appraisal tools for use in JBI Systematic Reviews) (22). The evaluation criteria consisted of 10 items, each with three possible judgments: “yes,” “no,” or “unclear.” ZYL and ZXT independently conducted the quality assessment, and any contentious studies were discussed with a third researcher (CF) to determine the final decision. Studies were categorized as low quality if the proportion of “yes” responses to the 10 evaluation criteria was below 60%; medium quality if the proportion was between 70% and 90%; and high quality if all items received a “yes” response (23).

### 2.6 Data extraction and synthesis

Two researchers (ZYL & ZXT) independently extracted data from the included studies using the JBI meta-aggregation approach (22). Data synthesis followed the JBI meta-aggregation approach, involving three stages. First, individual findings were extracted and classified as unequivocal or credible. Second, findings with similar meanings were grouped into categories. Third, these categories were further synthesized into overarching themes to generate actionable synthesized findings. This approach prioritizes transparency and applicability rather than theoretical reinterpretation. A finding was rated as unequivocal if it was accompanied by directly corresponding participant verbatim quotes, had no plausible alternative interpretation, and was stated using definitive language by the authors. A finding was rated as credible if it was supported by exemplar quotes but involved a moderate degree of abstraction or used qualifiers such as “may” or “sometimes.” This method categorized the extracted results from each study based on thematic similarity and further synthesized these categories. The extracted data included authorship, country of publication, year of publication, participant characteristics (number, age), study objectives, primary research methods, and reported barriers and facilitators. ZYL and ZXT conducted the data synthesis analysis. The extracted facilitators and/or barriers were organized into descriptive main themes and sub-themes through an inductive approach.

## 3 Results

### 3.1 Searching results

**Figure 1:**
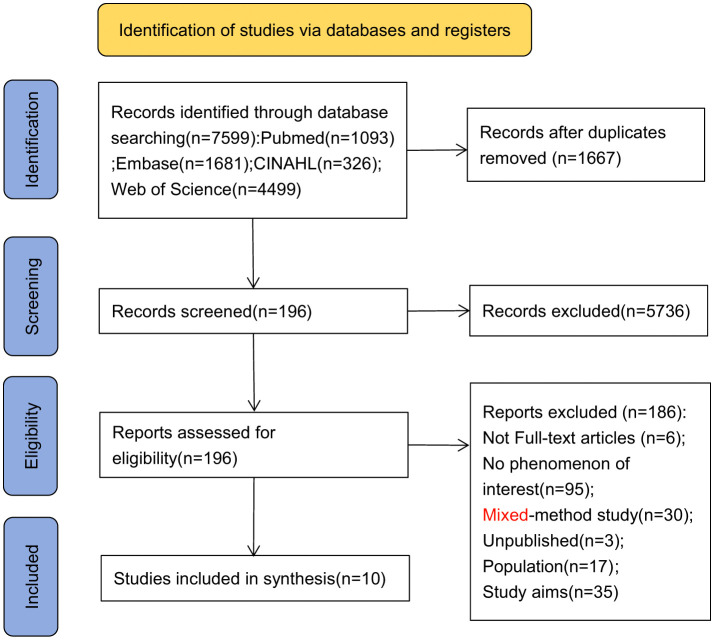
PRISMA flowchart for literature search.

As shown in Figure 1, a total of 7,599 articles were identified through the search. After removing duplicates, 5,932 articles were screened by title and abstract, resulting in the exclusion of 5,736 articles. Finally, 196 full-text articles were reviewed, and 10 qualitative studies were included in this systematic review. The complete list of included studies is provided in Supplementary File 3.

### 3.2 Study characteristics

Table 1 provides a description of the included studies. Among the 10 qualitative studies included, two used focus group methods, three employed semi-structured interviews, four used in-depth interviews, and one combined in-depth interviews with focus groups. Of the 10 included studies, three were conducted in the United States, one in India, and the remaining six in African countries, specifically Uganda, Tanzania, Zimbabwe, Ethiopia, South Africa, and Côte d'Ivoire. Across all 10 studies, a total of 226 WLWH were included, with the mean age ranging from 20 to 62 years.

**Table 1:** Characteristics of studies included.

References	Country	Aim	Characteristic of participants (sample size, age)	Methodological & sampling approach	Method of data collection and analysis
Andrasik et al. (24)	United States	Focus on how women who live in a severely economically depressed and racially segregated neighborhood experience barriers to cervical cancer screening.	*N* = 35 age (y): mean = 36.86 (SD ± 7.43)	•Qualitative design; •Convenience sampling	•Individual semi-structured interviews; •Qualitative data analysis
Fletcher et al. (25)	United States	To describe barriers and facilitators related to cervical cancer screening in a sample of women living with HIV (WLWH) seeking care at an integrated HIV clinic in Houston, Texas.	*N* = 33 age (y): mean = 51.0 (SD ± 8.78)	•Inductive qualitative design; •Convenience sampling	•Focus group sessions; •Inductive content analysis
Williams et al. (26)	United States	To examine sociocultural and structural factors associated with cervical cancer screening among HIV-positive African Americans in Alabama	*N* = 20 age (y): mean = 49(28–62)	•Qualitative design; •Convenience sampling	•In-depth interviews; •Content analysis
Bukirwa et al. (27)	Uganda	This study assessed factors associated with cervical screening uptake among HIV infected women at Mildmay Uganda	*N* = 18 age (y): above 25	•Cross-sectional qualitative design; •Convenience sampling	•In-depth interviews; •Content analysis
Kung et al. (28)	India	To evaluated individual and interpersonal factors influencing cervical cancer screening among WLWH in Surat, India.	*N* = 25 age (y): mean= 37.2 (SD ± 6.1)	•Qualitative design; •Purposeful sampling	•In-depth interviews; •Directed content analysis
Matenge and Mash (32)	South Africa	To explore the barriers to women with HIV accessing cervical cancer screening in Kgatleng district, Botswana	N=14 age (y): above 21	•Phenomenological qualitative design; •Purposive sampling	•Semi-structured interviews; •Qualitative data analysis
Bateman et al. (29)	Tanzania	To identify barriers and facilitators to cervical cancer screening, diagnosis, follow-up care, and treatment among human immunodeficiency virus (HIV)-infected women and clinicians and to explore the acceptability of patient navigators in Tanzania.	*N* = 19 age (y):(24–57)	•Qualitative design; •Convenience sampling	•Focus group sessions; •Thematic analysis
Mensah et al. (17)	Africa	To assess the preintervention acceptability of HPV screening among women living with HIV (WLWH) in Abidjan, Côte d'Ivoire.	*N* = 12 age (y):(25–55)	•Qualitative design; •Convenience sampling	•Semidirected interviews; •Inductive content analysis
Mpata and Nkosi (31)	Zimbabwe	Aim at describing the experiences of screening for cervical cancer and motivation behind screening.	*N* = 36 age (y): above 20	•Qualitative design; •Purposivecriterion based sampling	•In-depth interviews •focus group sessions; •Descriptive method of data analysis
Kebede et al. (30)	Ethiopia	To investigate cervical cancer screening barriers among WLWH at Yekatit 12 Hospital Medical College, Ethiopia, 2021.	*N* = 14 age (y):(22–49)	•Phenomenological qualitative design; •Purposive sampling	•In-depth interviews; •Thematic analysis

### 3.3 Methodological quality and level of dependability

As shown in Table 2, all studies scored highly on participant representation (item 8) and on the conclusion that the results reported in the studies were derived from data analysis or interpretation (item 10). Item 8 reflects the adequacy of participant representation, while Item 10 assesses the alignment between data interpretation and conclusions, both of which are critical indicators of qualitative rigor. All studies demonstrated good consistency between the research methods and the research objectives. Three of the included studies were rated as high quality, while the remaining seven were rated as medium quality, no low-quality studies were identified.

**Table 2:** Summary of the methodological quality.

References	1	2	3	4	5	6	7	8	9	10	Overall	Total percent “yes” response	Dependability rating
Andrasik et al. (24)	Y	Y	Y	Y	Y	Y	N	Y	Y	Y	B	90%	Moderate
Fletcher et al. (25)	Y	Y	Y	Y	Y	N	N	Y	Y	Y	B	80%	Moderate
Williams et al. (26)	Y	Y	Y	Y	Y	Y	N	Y	Y	Y	B	90%	Moderate
Bukirwa et al. (27)	Y	Y	Y	Y	Y	N	N	Y	Y	Y	B	80%	Moderate
Matenge and Mash (32)	Y	Y	Y	Y	Y	Y	N	Y	Y	Y	B	90%	Moderate
Kung et al. (28)	Y	Y	Y	Y	Y	Y	Y	Y	Y	Y	A	100%	High
Bateman et al. (29)	Y	Y	Y	Y	Y	Y	Y	Y	Y	Y	A	100%	High
Mensah et al. (17)	Y	Y	Y	Y	Y	N	N	Y	Y	Y	B	80%	Moderate
Mpata and Nkosi (31)	Y	Y	Y	Y	Y	N	N	Y	Y	Y	B	80%	Moderate
Kebede et al. (30)	Y	Y	Y	Y	Y	Y	Y	Y	Y	Y	A	100%	High

From the 10 studies included in the meta-aggregation, a total of 106 results were rated as “credible” or “clear.” The 106 study results were aggregated into six categories, which were then synthesized into two overarching results. We treated every findings equally. The process of generating and summarizing the categories of facilitators and barriers to cervical cancer screening among WLWH is detailed in Supplementary File 4.

The item of Critical Appraisal tools for use in JBI Systematic Reviews: (1) Is there congruity between the stated philosophical perspective and the research method? (2) Is there congruity between the research methodology and the research question or objectives? (3) Is there congruity between the research methodology and the methods used to collect data? (4) Is there congruity between the research methodology and the representation and analysis of data? (5) Is there congruity between the research methodology and the interpretation of results? (6) Is there a statement locating the researcher culturally or theoretically? (7) Is the influence of the researcher on the research, and vice- versa, addressed? (8) Are participants, and their voices, adequately represented? (9) Is the research ethical according to current criteria or, for recent studies, and is there evidence of ethical approval by an appropriate body? (10) Do the conclusions drawn in the research report flow from the analysis, or interpretation, of the data?

Note: “Y” means “Yes,” “N” means “No”.

A study was rated as “A” if all checklist items were answered “Yes,” and as “B” if one or more items were answered “No.” An “A” rating indicates higher study quality and lower risk of bias, whereas a “B” rating indicates moderate or lower study quality with some risk of bias.

### 3.4 Meta-aggregation

#### 3.4.1 Synthesized results 1: driving forces: intrinsic and extrinsic motivators for cervical cancer screening among WLWH

**Figure 2:**
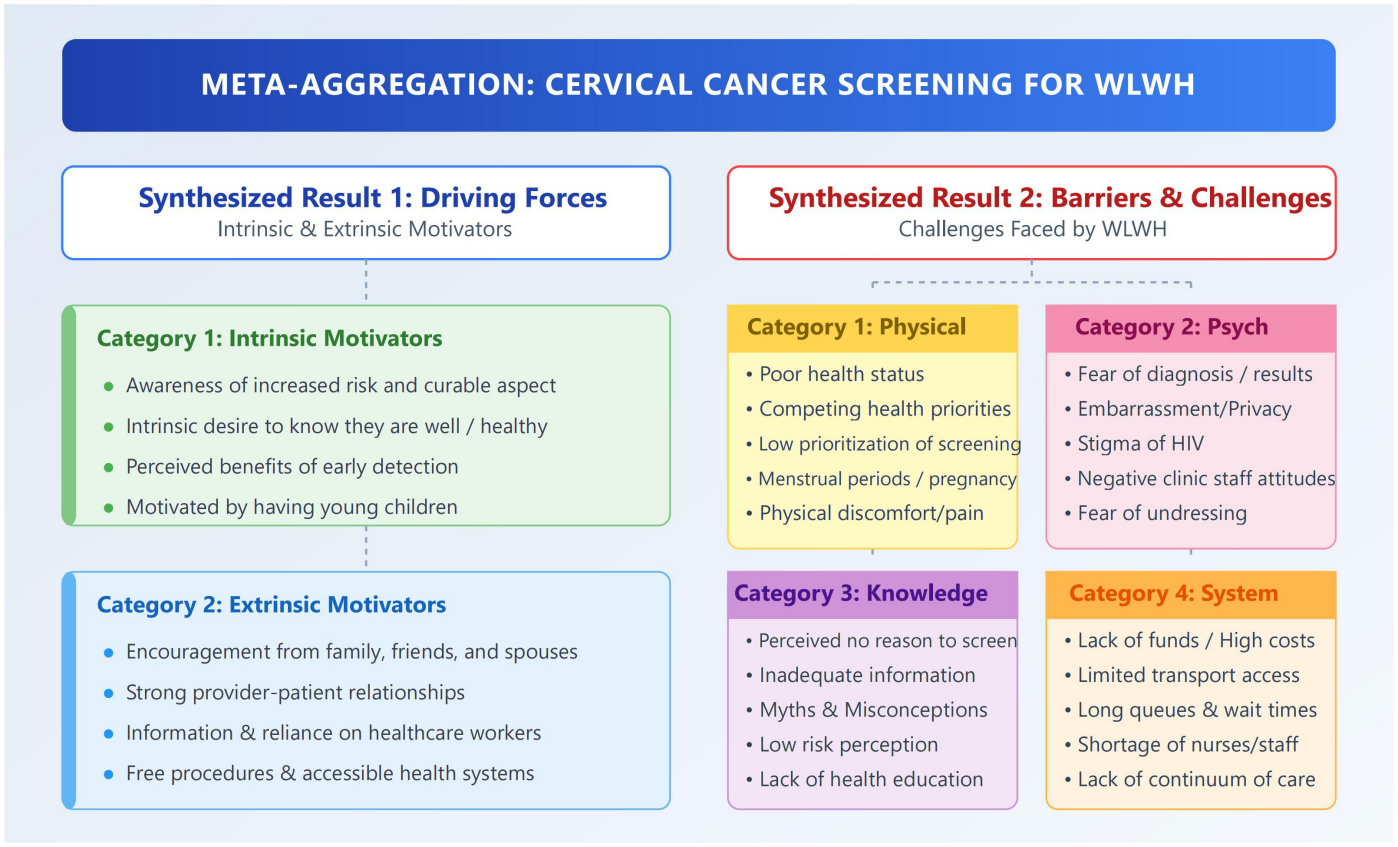
Scheme of the synthesized results.

As shown in Figure 2, in exploring the facilitators for cervical cancer screening among WLWH, the research identifies both intrinsic and extrinsic motivators that significantly influence their participation in these health programs. Sixteen synthesized statements supported the intrinsic motivators, and 11 synthesized statements supported the extrinsic motivators. Intrinsic motivators include personal awareness of health risks and the curability of cervical cancer, the desire for health maintenance and future planning, and the responsibilities of motherhood. Extrinsic motivators encompass the support from family and spouses, positive healthcare provider interactions, and the alleviation of financial barriers.

##### Intrinsic motivators

1. Awareness of Risk and Curability: Many women recognize their increased risk of cervical cancer due to HIV status, which naturally leads to a proactive approach toward screening. The realization that cervical cancer is treatable if detected early further alleviates fears and motivates these women to seek preventive care (17, 25, 30).
2. Health Maintenance and Future Planning: The desire to maintain good health and ensure a future for themselves and their families is a strong motivator. Women express a sense of duty and urgency to remain healthy not just for themselves but also to continue caring for their loved ones (27, 28).
3. Family Responsibilities: Motherhood intensifies the motivation for screening. Knowing that their health directly affects their children's future, many women are compelled to participate in screening programs to ensure they remain healthy and active in their children's lives (31).

##### Extrinsic motivators

1. Family and Spousal Support: Emotional and logistical support from family and spouses significantly influences women's decisions to undergo screening. This support ranges from encouragement to accompanying them to screening appointments, which not only eases logistical burdens but also provides emotional reassurance (26, 28, 31).
2. Healthcare Provider Support: Positive interactions with empathetic healthcare providers boost women's confidence in the screening process. Clear communication and gentle handling during screenings make the experience more comfortable and less intimidating, thereby increasing their likelihood to engage in regular screenings (17, 25).
3. Financial Support: The provision of free screening services removes a significant barrier—cost. With financial constraints lifted, women are more likely to opt for screening services, knowing that they can access these essential health services without the burden of expense (17).

These factors highlight the complex interplay of personal motivation, social support, and institutional facilitation in promoting cervical cancer screening. They collectively enhance the likelihood that WLWH will participate in screening programs, crucial for early detection and treatment.

#### 3.4.2 Synthesized results 2: barriers to cervical cancer screening: challenges faced by WLWH

Cervical cancer screening is crucial for early detection and treatment, yet WLWH face numerous barriers that can impede their participation in such programs. These barriers can be categorized into physical impairments (3 synthesized statements supported), psychological and emotional challenges (19 synthesized statements supported), perceptual and knowledge deficits (22 synthesized statements supported), and inadequacies in social support systems (35 synthesized statements supported). Each category encapsulates specific challenges that collectively hinder the screening uptake among this vulnerable group.

##### Physical impairments

Physical health issues associated with HIV can deter women from participating in cervical cancer screening:

1. Dermatological Issues: Many WLWH report experiencing severe skin conditions that affect their self-esteem and comfort in clinical settings. As one participant noted, “My skin was looking bad” (27). Such visible signs of illness can lead to self-consciousness, discouraging them from seeking medical care.

2. Chronic Conditions: Symptoms like significant weight loss and hypertension can complicate the screening process. One participant expressed, “I had even lost weight. Even my blood pressure was high” (27). These health concerns can make the physical experience of screening uncomfortable and intimidating.

3. Menstrual Complications: The timing of screenings can be problematic for women who experience irregular menstrual cycles. Some participants noted that they would avoid screening when close to their menstrual periods, as healthcare providers often advise against screening during that time. This leads to missed appointments and delays in necessary care (27).

##### Psychological and emotional challenges

The psychological burden of living with HIV significantly impacts women's willingness to engage with health services:

1. Fear of Diagnosis: Many women harbor a deep-seated fear of receiving bad news during screenings. One participant stated, “I don't want to find out no more bad news” (26). “…and also if they tell me additional problem on current illness I did not able to survive because of stress” (30). This apprehension can lead to avoidance of screening altogether, as the thought of an additional serious diagnosis feels overwhelming.

2. Privacy Concerns: The intimate nature of cervical cancer screenings can provoke anxiety, particularly for women who feel vulnerable due to their HIV status. Concerns about exposure and privacy during the screening process are common. As one woman expressed, “How can I expose my privacy to a young person for screening?” (27). “I don't want anybody else to look at my own body or touch my own body, that's my own” (30).

3. Stigma and Shame: The stigma attached to being HIV-positive can exacerbate feelings of shame and isolation. Many women fear discrimination and judgment from both healthcare providers and the community. One participant shared, “There's a lot of stigma... people will treat you differently” (26). This fear can lead to reluctance in seeking necessary medical care.

##### Perceptual and knowledge deficits

A lack of accurate information about cervical cancer and its risks contributes to lower screening rates:

1. Misconceptions about Risk: Some women believe that without a family history of cancer, they are not at risk. One participant noted, “I don't have any family members with cancer... so I won't catch it” (26). This lack of awareness regarding the risks associated with HIV can lead to underestimating their need for screening.

2. Skepticism about Treatment: Many women express doubt about the effectiveness of cervical cancer treatments, which can further deter them from seeking early diagnosis. One participant mentioned, “They say that if you're screened early you can be cured... I don't know” (17). “… this disease is less likely to be cured with medicine once developed, no need of screening as I heard from other people that almost all die once they develop the cervical cancer” (30).This skepticism can diminish their motivation to undergo screening.

##### Social support system inadequacies

Inadequate support systems critically reduce screening participation:

- Economic Barriers: Many women face financial challenges that prevent them from accessing screening services. One participant lamented, “I don't have any income... I don't have the $5 to fill out the paperwork” (32). “… because of the high cost of screening and treatment. I do not wish to use the cervical cancer screening service …” (30). The costs associated with screening can be prohibitive, leading to delays or avoidance of necessary care.

2. Healthcare System Barriers: Negative experiences within the healthcare system can discourage women from returning for screenings. Issues such as long waiting times and painful procedures significantly affect their willingness to participate. One woman commented, “Pap smears I hate them. I know it hurts because I done been there” (26).

3. Lack of Continuous Support: Many women feel that the healthcare system does not provide the consistent and supportive care they need. As noted by a participant, “I don't want a different person every time I'm there” (32). This inconsistency can lead to feelings of neglect and a decreased likelihood of returning for follow-up screenings.

## 4 Discussions

This review provides a synthesis of qualitative evidence on facilitators and barriers to cervical cancer screening among WLWH. While consistent patterns were identified, the findings extend beyond simple description by highlighting the interplay between individual perceptions, social relationships, and healthcare system structures. Compared with previous quantitative studies, which primarily focus on demographic and socioeconomic predictors, our findings emphasize the importance of psychosocial factors such as stigma, fear, and relational support. This suggests that interventions targeting behavioral and emotional dimensions may be as critical as improving structural access. However, the interpretive nature of this synthesis should be considered. The reliance on meta-aggregation prioritizes transparency and applicability but may limit deeper theoretical insights compared to more interpretive approaches.

The synthesized results demonstrate that intrinsic motivation among WLWH is a key factor in promoting cervical cancer screening. Studies have found that there is a certain correlation between HIV infection and cervical cancer, which may be due to the abnormal proliferation of the cervix caused by HIV infection, thereby increasing the risk of cervical cancer (33). In fact, some studies have emphasized that women living with HIV (WLWH) show a willingness to participate in screening after obtaining sufficient information about the risk of cervical cancer infection, or they are further motivated to seek preventive health services if they recognize the curability of cervical cancer at an early stage (34–36). This suggests that awareness alone may not directly lead to screening uptake; rather, it is the perceived personal relevance and perceived severity of risk that translate knowledge into action. For example, HPV testing, as one of the important methods for cervical cancer screening, can help women detect HPV infection early, while liquid-based cytology (LBC) testing improves the detection rate of precancerous cells, achieving early detection, diagnosis, and treatment (37). The scientific and effective nature of these screening methods further encourages WLWH to actively participate in cervical cancer prevention and screening. A possible explanation is that WLWH choose to undergo screening only after recognizing the benefits of testing. Studies have pointed out that enhancing awareness and education can strengthen women's behavior in seeking health services, especially cervical cancer-related services (such as testing) (38, 39), which is consistent with the findings of Drokow's study (40). While previous studies have also highlighted the role of awareness, our findings further suggest that awareness operates through shaping risk perception and perceived benefits, rather than acting as an isolated determinant. In some studies, infected individuals expressed their sense of responsibility for maintaining health, such as the need to stay healthy and plan for the future (27, 28). In Kuhn's study, cervical cancer screening and treatment using human papillomavirus DNA testing significantly reduced cervical intraepithelial neoplasia among WLWH within 36 months (41), which is consistent with the conclusion of Boddu's study, indicating that participation in early cervical cancer screening methods such as HPV testing, visual inspection with acetic acid (VIA), and Pap smear cytology not only increases the detection rate among WLWH but also potentially improves the early cure rate of cervical cancer through treatment, thereby extending survival (10). Meanwhile, family responsibilities also enhance the screening motivation of WLWH to some extent (31). Maher's study pointed out that the enhanced sense of maternal responsibility makes women with children more likely to actively address health issues (42). Therefore, after becoming mothers, their motivation to participate in cervical cancer screening programs may be higher, especially when they realize that their health status is directly related to the future of their children (31). This is further emphasized in the importance of cervical cancer screening, as early screening can significantly improve the cure rate, and regular screening is an important step in protecting one's health.

In promoting the participation of WLWH in cervical cancer screening, external motivational factors, such as emotional support from family, spouses, and peers, play an important role (26, 28, 31, 43). Assefa's study showed that WLWH with partner support were 4.7 times more likely to undergo cervical cytology (CC) screening compared to those without partner support (44). This was further confirmed by Ogunwale's study, which identified male partner support as a key facilitator for WLWH to regularly undergo Pap tests (45). Our synthesis revealed that peer support (i.e., mutual support among friends who are also HIV-infected) can mitigate disease-related stigma through collective participation, build confidence, and create a positive environment for cervical cancer screening (43). This aligns with Mboineki's study, which found that peer-led navigation strategies help HIV-negative women overcome screening barriers, significantly enhancing their willingness and acceptance of screening, thereby increasing screening rates (46). Additionally, support from healthcare providers significantly improves the utilization of cervical cancer screening services among WLWH, consistent with Gebrekirstos's findings (47). On one hand, screening information provided by healthcare providers offers necessary medical guidance for women living with HIV (WLWH), prompting them to schedule timely examinations and understand the screening process and its importance (48). Peterson's study emphasized the importance of effective communication related to cervical cancer screening, encouraging healthcare providers to actively engage with patients through information sharing and shared decision-making to alleviate women's fears and concerns about the screening process, thereby increasing cervical cancer screening rates (49). On the other hand, Yen's study found that reducing the pain associated with Pap smear screening can enhance women's willingness to undergo regular Pap tests, serving as an effective means to promote women's participation in cervical cancer screening (50). From a financial perspective, providing convenient and affordable screening services for WLWH helps them overcome economic barriers (17). This echoes Sigfrid's previous research, which indicated that distributing test kits and establishing infrastructure can provide more support to reduce logistical barriers and increase the introduction of cervical cancer screening (51).

In primary care, HIV-related health issues constitute a major barrier to cervical cancer screening (29). Due to compromised immunity, people living with HIV/AIDS experience various physical symptoms, including muscle and joint pain, fatigue, poor sleep quality, and abdominal pain. Previous studies have shown that the presence of these non-gynecological symptoms is a barrier to cervical cancer screening among WLWH (52, 53). These physical factors were also confirmed in our synthesis as major barriers to screening participation among WLWH, possibly because women infected with HIV may choose to neglect the health needs of cervical cancer screening due to physical weakness caused by symptom burden. Additionally, skin problems caused by the disease have been identified as an additional barrier to cervical cancer screening for WLWH (27). Reduced self-esteem and decreased comfort due to skin damage may lead some women to be unwilling to seek medical help. The association between self-esteem and the behavior of seeking health help has been proven, which may be a key factor causing such barriers (54). Furthermore, healthcare providers could offer special cervical cancer screening information for women during pregnancy, such as screening for pregnant women between 16 and 20 weeks of gestation using cytology and HPV testing to increase screening coverage (55). It is also recommended to optimize the screening appointment process to facilitate the participation of WLWH in cervical cancer screening at different physiological stages.

In the psychological dimension, WLWH exhibit fear and anxiety regarding cervical cancer screening. In many countries, more than 250,000 women die from cervical cancer each year due to limited or severe lack of treatment (56). In most studies expressing screening fear, the fear of diagnosis is closely related to women's fear of death (17, 24, 26, 28). This phenomenon is not only observed in WLWH but also reported in non-WLWH. Abdi's study pointed out that women avoid screening because cancer at an advanced stage is seen as an excuse for death (57). Similarly, the results of this review indicate that concerns about privacy exposure may be a barrier to cervical cancer screening for WLWH (27, 58). For example, the gender of healthcare professionals often leads to a lack of security among women, making them unwilling to expose private areas for cervical cancer screening (59). This is similar to a cross-sectional study in Canada (*N* = 1,189) among women living with HIV (WLWH), which showed that the presence of male caregivers is one of the main factors for delayed cervical cancer screening (60). Given the limited availability of female healthcare providers for WLWH, some studies have proposed that self-sampling is an acceptable and promising screening method (61). Additionally, negative experiences that women living with HIV (WLWH) encounter in healthcare settings may lead to shame and hinder their subsequent adherence to cervical cancer screening (26). Studies have described women's experiences in breast and cervical cancer screening, indicating that they felt discrimination and stigmatization from healthcare workers during screening, which negatively impacted their behavior in seeking healthcare (62). It is recommended that healthcare providers and health professionals offering screening receive more health education on services for WLWH to reduce such barriers.

This review reveals that inadequate health knowledge among WLWH is one of the important factors affecting the effectiveness of cervical cancer screening, highlighting the core role of accessible and easily understandable health education measures in promoting cervical cancer screening (63). In fact, multiple studies have pointed out that if participants lack awareness of cervical cancer risk, they may mistakenly believe that without a family history of cancer, they are not at risk of cervical cancer (25, 26, 64). Additionally, some studies have shown that the public's skepticism about the curability of cervical cancer leads to generally low acceptance of cervical cancer screening (17, 32). Particularly, studies have shown that fatalism, cultural taboos, and the negative notion in religious beliefs that cancer is an inevitable fate all hinder women's participation in cervical cancer screening, emphasizing the necessity for health professionals to provide comprehensive health education for women from different cultural backgrounds (65).

Studies have indicated that the inadequacy of social support systems constitutes a potential barrier, within which factors related to the healthcare system are frequently mentioned. The included studies show that prolonged clinical waiting times are one of the main barriers in the cervical cancer screening process (25, 27, 31). A study quantifying the time costs for 105 women across six clinics in the United States during cervical cancer screening revealed that the average waiting room time for patients was 16.9 min, accounting for 25% of the total cost of cervical cancer screening (66). Interventions prioritizing the training of healthcare personnel have been proven to reduce long waiting times and create a more efficient and patient-centered healthcare experience (67). It is recommended to provide comprehensive medical training for healthcare personnel to enhance their cervical cancer screening skills, which may improve the knowledge and skill base of healthcare providers while reducing waiting times to promote WLWH's participation in screening healthcare. Another recurring determinant is financial barriers; economic challenges may pose a significant obstacle to cervical cancer screening for low-income women (68, 69). In many areas, WLWH face financial difficulties, and cervical cancer screening, as a preventive service, is often considered a secondary need compared to basic living requirements. They may refuse to participate in cervical cancer screening if they have to pay for the tests (17, 24, 70). Although some low- and middle-income countries offer free cervical cancer screening services, additional costs such as transportation fees may still act as barriers for women to receive screening (71, 72). Therefore, when educating WLWH about the importance of cervical cancer and its screening, we need to emphasize the rational planning of expenses and ensure effective financial coverage for screening costs. It is also important to clearly communicate when clinics offer free cervical cancer screening.

## 5 Implications for practice and policy

This synthesis identified three previously underemphasized facilitators with direct implications for practice. First, women living with HIV (WLWH) prioritize relational continuity with a known provider over generic communication skills or provider gender. Consequently, cervical cancer screening should be integrated into existing HIV chronic care teams rather than referring women to unfamiliar gynecology services. Second, peer navigation delivered by WLWH who have completed screening reduces stigma more effectively than didactic instruction alone. Third, motherhood represents a positive, future-oriented motivator, not merely fear of death, suggesting that health messages should reframe screening as an act of protecting one's family. Based on these findings, we propose resource-stratified strategies. In resource-limited settings: embed screening into routine HIV visits, train screened peers as navigators, and expand HPV self-sampling to address privacy and provider-gender barriers. In resource-rich settings: use electronic health records to track continuity navigators, establish reimbursed peer programs, and implement expedited appointment pathways to reduce waiting times. Across both contexts, eliminating out-of-pocket costs and providing transportation subsidies are essential. These recommendations move beyond traditional awareness-raising toward relationship-based, peer-modeled, and motivationally-tailored interventions.

## 6 Limitations and prospects

Although the study sufficiently ensured the rigor of a systematic review of qualitative research, there are still some limitations. First, the availability of high-quality studies on barriers to cervical cancer screening among WLWH is limited, and further high-quality research is needed to explore the barriers and facilitators to cervical cancer screening in this high-risk population. Most of the included studies were rated “No” on Items 6 and 7, indicating a lack of adequate reflexivity. Specifically, the potential biases of the researchers and the interactive influences during data collection and analysis were not systematically examined. We recommend that future studies adhere to the COREQ or SRQR guidelines to report the reflexive process in detail. Moreover, with only 10 studies and 226 participants, our ability to examine variation across diverse healthcare systems, geographic regions, or sub-groups is limited. Another limitation to consider is that the search excluded studies not written in English. Therefore, it is possible that some studies were overlooked and not included in the review. Furthermore, the ways in which individual primary studies reported cross-cutting factors and the level of detail provided varied considerably. Thus, this review was unable to conduct subgroup analyses, which also reflects the current underappreciation of intersectionality perspectives in the research field. Finally, most of the studies included in this systematic review were conducted in developing countries, with only three in developed countries, so there may be significant research gaps between countries or regions.

## 7 Conclusions

This review identifies key barriers and facilitators to cervical cancer screening among WLWH, including individual factors (risk perception, understanding of health, family responsibilities, trust in HIV care), systemic barriers (symptom burden, fear, privacy concerns, gaps in knowledge, negative healthcare experiences, financial, and logistical constraints), and social factors (stigma, socioeconomic status). To improve screening uptake, strategies should focus on reducing fear and stigma, enhancing awareness, addressing financial and structural barriers, and fostering supportive healthcare relationships. Future research should explore these factors in diverse settings, especially in developed countries, to inform policies that prioritize vulnerable populations and integrate cervical cancer screening into routine HIV care.
