# Supplementary material for: Facilitators and barriers to cervical cancer screening among women living with HIV: a systematic review of qualitative studies
Source: Front Public Health. 2026 Jun 22;14:1809112. doi: 10.3389/fpubh.2026.1809112 (PMC13333709; doi:10.3389/fpubh.2026.1809112)
Supplement: Supplementary file 1 [file Data_Sheet_1.docx]

**Supplementary file-2 Searching strategies**

| **Pubmed** |  |  |
| --- | --- | --- |
| Search number | Query | Results |
| 1 | HIV[MeSH Terms] AND (2000:2024[pdat]) | 77,380 |
| 2 | AIDS[MeSH Terms] AND (2000:2024[pdat]) | 22,849 |
| 3 | ((HIV[Title/Abstract]) OR (AIDS[Title/Abstract]) OR (Acquired immunodeficiency syndrome[Title/Abstract]) OR (Acquired immune deficiency syndrome[Title/Abstract]) OR (Human immunodeficiency[Title/Abstract])) AND (2000:2024[pdat]) | 355,695 |
| 4 | #1 OR #2 OR #3 | 361,242 |
| 5 | ((women[Title/Abstract]) OR (woman[Title/Abstract]) OR (women[Title/Abstract])) AND (2000:2024[pdat]) | 1,201,711 |
| 6 | cervical smear[MeSH Terms] AND (2000:2024[pdat]) | 10,712 |
| 7 | ((Cervical screening[Title/Abstract]) OR (pap[Title/Abstract]) OR (Papanicolaou test[Title/Abstract]) OR (cervical smear[Title/Abstract]) OR (early detection of cancer[Title/Abstract]) OR (neoplasm[Title/Abstract]) OR (human papilloma virus[Title/Abstract])) AND (2000:2024[pdat]) | 98,734 |
| 8 | #6 OR #7 | 103,688 |
| 9 | #4 AND #5 AND #8 | 1,093 |

| **WOS** |  |  |
| --- | --- | --- |
| Search number | Query | Results |
| 1 | (TI=(HIV) OR TI=(AIDS) OR TI=(Acquired immunodeficiency syndrome) OR TI=(Acquired immune deficiency syndrome) OR TI=(Human immunodeficiency)) AND (DOP=(2000-01-01/2024-12-31)) | 572,413 |
| 2 | ((AB=(HIV) OR AB=(AIDS) OR AB=(Acquired immunodeficiency syndrome) OR AB=(Acquired immune deficiency syndrome) OR AB=(Human immunodeficiency))) AND (DOP=(2000-01-01/2024-12-31)) | 1,319,282 |
| 3 | #1 OR #2 | 1,508,159 |
| 4 | (TI=(female) OR TI=(woman) OR TI=(women))AND (DOP=(2000-01-01/2024-12-31)) | 929,706 |
| 5 | (AB=(female) OR AB=(woman) OR AB=(women) )AND (DOP=(2000-01-01/2024-12-31)) | 3,584,410 |
| 6 | #4 OR #5 | 3,866,776 |
| 7 | (TI=(Cervical screening) OR TI=(pap) OR TI=(Papanicolaou test) OR TI=(cervical smear) OR TI=(early detection of cancer) OR TI=(neoplasm) OR TI=(human papilloma virus) )AND (DOP=(2000-01-01/2024-12-31)) | 75,415 |
| 8 | (AB=(Cervical screening) OR AB=(pap) OR AB=(Papanicolaou test) OR AB=(cervical smear) OR AB=(early detection of cancer) OR AB=(neoplasm) OR AB=(human papilloma virus) )AND (DOP=(2000-01-01/2024-12-31)) | 275,851 |
| 9 | #7 OR #8 | 310,175 |
| 10 | #3 AND #6 AND #9 | 4,499 |

| **Embase** |  |  |
| --- | --- | --- |
| Search number | Query | Results |
| 1 | hiv:ab,ti OR aids:ab,ti OR 'acquired immunodeficiency syndrome':ab,ti OR 'acquired immune deficiency syndrome':ab,ti OR 'human immunodeficiency':ab,ti | 474,893 |
| 2 | female:ab,ti OR woman:ab,ti OR women:ab,ti | 2901463 |
| 3 | cervical screening':ab,ti OR pap:ab,ti OR 'papanicolaou test':ab,ti OR 'cervical smear':ab,ti OR 'early detection of cancer':ab,ti OR neoplasm:ab,ti OR 'human papilloma virus':ab,ti | 131899 |
| 4 | #1 AND #2 AND #3 | 1681 |

| **CINAHL** |  |  |
| --- | --- | --- |
| Search number | Query | Results |
| 1 | AB(HIV OR AIDS OR "Acquired immunodeficiency syndrome" OR "Acquired immune deficiency syndrome" OR "Acquired immune deficiency syndrome" OR "Human immunodeficiency") | 85,419 |
| 2 | AB(female OR woman OR women) | 608,990 |
| 3 | AB(“Cervical screening" OR pap OR "Papanicolaou test" OR "cervical smear" OR "early detection of cancer" OR "neoplasm" OR "human papilloma virus") | 13,784 |
| 4 | #1 AND #2 AND #3 | 326 |
