# Supplementary material for: Facilitators and barriers to cervical cancer screening among women living with HIV: a systematic review of qualitative studies
Source: Front Public Health. 2026 Jun 22;14:1809112. doi: 10.3389/fpubh.2026.1809112 (PMC13333709; doi:10.3389/fpubh.2026.1809112)
Supplement: Supplementary file 2 [file Data_Sheet_2.docx]

**Supplementary file-3 full included studies**

1.Andrasik MP, Rose R, Pereira D, Antoni M. Barriers to cervical cancer screening among low-income HIV-positive African American women. J Health Care Poor Underserved. 2008;19(3):912-25.

2.Fletcher FE, Buchberg M, Schover LR, Basen-Engquist K, Kempf MC, Arduino RC, et al. Perceptions of barriers and facilitators to cervical cancer screening among low-income, HIV-infected women from an integrated HIV clinic. AIDS Care. 2014;26(10):1229-35.

3.Michelle W, Linda M, Mirjam-Colette K, Eric C, Isabel S. Structural and sociocultural factors associated with cervical cancer screening among HIV-infected African American women in Alabama. AIDS patient care and STDs. 2015;29(1):13-9.

4.Bukirwa A, Mutyoba JN, Mukasa BN, Karamagi Y, Odiit M, Kawuma E, et al. Motivations and barriers to cervical cancer screening among HIV infected women in HIV care: a qualitative study. BMC Womens Health. 2015;15:82.

5.Matenge TG, Mash B. Barriers to accessing cervical cancer screening among HIV positive women in Kgatleng district, Botswana: A qualitative study. PLoS One. 2018;13(10):e0205425.

6.Kung TH, Gordon JR, Abdullahi A, Barve A, Chaudhari V, Kosambiya JK, et al. "My husband says this: If you are alive, you can be someone…": Facilitators and barriers to cervical cancer screening among women living with HIV in India. Cancer Causes Control. 2019;30(4):365-74.

7.Bateman LB, Blakemore S, Koneru A, Mtesigwa T, McCree R, Lisovicz NF, et al. Barriers and Facilitators to Cervical Cancer Screening, Diagnosis, Follow-Up Care and Treatment: Perspectives of Human Immunodeficiency Virus-Positive Women and Health Care Practitioners in Tanzania. Oncologist. 2019;24(1):69-75.

8.Mensah K, Assoumou N, Duchesne V, Pourette D, DeBeaudrap P, Dumont A. Acceptability of HPV screening among HIV-infected women attending an HIV-dedicated clinic in Abidjan, Côte d'Ivoire. BMC Womens Health. 2020;20(1):155.

9.Mpata PC, Nkosi ZZ. Experiences of cervical cancer screening in HIV-positive women in Zimbabwe. Curationis. 2021;44(1):e1-e7.

10.Kebede HA, Bekele GE, Gebrehiwot EM, Kitesa G, Derbew A. Cervical Cancer Screening Barriers Among HIV Positive Women Attending ART Clinic at Yekatit 12 Hospital Medical College, Addis Ababa, Ethiopia: A Qualitative Study. Journal of Family Medicine and Health Care. 2024;10(1):1-10.
