# Supplementary material for: Facilitators and barriers to cervical cancer screening among women living with HIV: a systematic review of qualitative studies
Source: Front Public Health. 2026 Jun 22;14:1809112. doi: 10.3389/fpubh.2026.1809112 (PMC13333709; doi:10.3389/fpubh.2026.1809112)
Supplement: Supplementary file 3 [file Data_Sheet_3.docx]

**Supplementary file-4 Summary of study finding, categories, and synthesized categories to generate synthesized findings on the facilitators and barriers to cervical cancer screening among HIV-positive women.**

| Synthesized results 1: Driving Forces: Intrinsic and Extrinsic Motivators for Cervical Cancer Screening among HIV-Positive Women | | |
| --- | --- | --- |
| Findings [Credibility Rating] | Categories | Synthesized Category |
| 13.Awareness of HIV-infected womens increased risk of cervical cancer | Awareness of Risk and Curability | Intrinsic Motivators |
| 30.Risk perceptions for cervical cancer |  |  |
| 62.Felt cervical smears were important |  |  |
| 77.Aware of the potential effect of HIV on cancer development |  |  |
| 83.Aware of the curable aspect of cervical cancer |  |  |
| 89.Felt that being HIV-positive made them more vulnerable to cervical cancer |  |  |
| 98.Being HIV positive is commonly thought as risk for getting cervical cancer |  |  |
| 19.An intrinsic motivation to know they are well | Health Maintenance and Future Planning |  |
| 36.They screened because they desired total good health and felt that knowing that they had the disease early would save their lives. |  |  |
| 50.The participants were aware that screening was the only way to know if one had cervical cancer |  |  |
| 69.Motivated to learn more information about cervical cancer and cervical cancer screening because they had a desire to stay healthy |  |  |
| 82.Perceived benefits |  |  |
| 84.The need to stay healthy |  |  |
| 87.Self-efficacy |  |  |
| 100.Perceived Benefits of Cervical Cancer Screening |  |  |
| 91.Having young children motivated her to screen | Family Responsibilities |  |
| 25.They received encouragement from family, friends, and  spouses | Family and Spousal Support | Extrinsic Motivators |
| 71.Interpersonal influences from family members |  |  |
| 90.Support and encouragement from the spouse |  |  |
| 15.Strong provider-patient relationships | Healthcare Provider Support |  |
| 23.The gender of the healthcare provider was no longer a factor |  |  |
| 72.Interpersonal interactions with healthcare workers |  |  |
| 73.Information provided by healthcare providers |  |  |
| 85.Healthcare workers were quoted as a resource to help women |  |  |
| 88.Rely on healthcare workers |  |  |
| 92.Access to information and having trust in the health delivery system |  |  |
| 86.The procedure was free | Financial Support |  |
| Synthesized results 2: Barriers to Cervical Cancer Screening: Challenges Faced by HIV-Positive Women | | |
| Findings [Credibility Rating] | Categories | Synthesized Category |
| 34.Poor health status of the women | Dermatological Issues | Physical Impairments |
| 35.Competing health priorities and low prioritization of cervical screening | Chronic Conditions |  |
| 37.Being in menstrual periods and being pregnant as hindrances to cervical screening | Menstrual Complications |  |
| 2.Many feared they might discover additional bad news if they saw a doctor. | Fear of Diagnosis | Psychological and Emotional Challenges |
| 21.A frequently reported barrier to getting Pap tests was the fear that the test would reveal an additional health problem. |  |  |
| 33.Feared pain during cervical cancer screening and other side effects of the procedure |  |  |
| 64.Feared the results |  |  |
| 70.Fear of cervical cancer screening and the results |  |  |
| 76.Cervical cancer is perceived as an additional burden after HIV |  |  |
| 78.Perceived barriers:Fear of a diagnosis |  |  |
| 24.Some women who find the Pap test embarrassing or who fear partner jealously are | Privacy Concerns |  |
| 39.Fear of invasion into their privacy |  |  |
| 40.The fear of undressing was a prominent issue |  |  |
| 102.Invasion of privacy and it is difficult for them to expose their private parts |  |  |
| 1.The shame and embarrassment of being HIV-positive was too much to bear | Stigma and Shame |  |
| 6.Negative past experiences |  |  |
| 26.The fear of stigma and discrimination |  |  |
| 49.Stigma |  |  |
| 60.Negative attitudes from clinic staff |  |  |
| 79.The stigma of HIV |  |  |
| 101.The fear associated with the test. |  |  |
| 106.Disrespect is one barrier to hinder to cervical screening |  |  |
| 3.Perceived no reason to go for screening | Misconceptions about Risk | Perceptual and Knowledge Deficits |
| 11.Inadequate information. |  |  |
| 20.Had misconceptions about the signs and symptoms of cervical cancer |  |  |
| 27.The participants did not believe that they were more susceptible to cervical cancer than HIV-uninfected women |  |  |
| 28.HIV-infected women stop having sex after they receive their HIV diagnosis and therefore feel that they do not need to get a Pap test |  |  |
| 29.Perceived severity. |  |  |
| 31.Risk perceptions for cervical cancer |  |  |
| 32.Myths and misconceptions about the process of screening |  |  |
| 47.Knowledge/Awareness |  |  |
| 48.Information Sources |  |  |
| 61.There was a general lack of knowledge about screening and cervical cancer in general |  |  |
| 63.New sensations in their body |  |  |
| 66.Lack of health education |  |  |
| 68.Lack of knowledge about cervical cancer and the Pap test |  |  |
| 81.Lack of knowledge |  |  |
| 93.Community awareness of cervical cancer screening |  |  |
| 96.Awareness on Cervical Cancer and Cervical Cancer Screening |  |  |
| 97.Awareness on Cause of Cervical Cancer |  |  |
| 14.Lack of awareness of cervical cancer as preventable disease | Skepticism about Treatment |  |
| 65.Not being aware that there is treatment for cervical cancer precursor lesions |  |  |
| 75.Perceived severity,cervical cancer is a deadly disease |  |  |
| 99.Cervical cancer is considered as a serious illness which can rapidly progressed and ends up with death irrespective of any medical intervention. |  |  |
| 4.Lack of funds | Economic Barriers | Social Support System Inadequacies |
| 5.Transportation. |  |  |
| 9.Conflicting needs |  |  |
| 16.Institutional barriers :Limited transportation access |  |  |
| 53.Contextual issues |  |  |
| 67.Alcohol misuse |  |  |
| 80.Lack of financial resources |  |  |
| 95.The high cost of treatment is an obstacle |  |  |
| 104.Cost of treatment |  |  |
| 7.Discomfort | Healthcare System Barriers |  |
| 10.Insufficient social support |  |  |
| 12.Pain and discomfort associated with receiving Pap smears and subsequent procedures |  |  |
| 17.Extensive wait time |  |  |
| 18.Systemic issues related to scheduling gynecological  Appointments |  |  |
| 22.The pain and discomfort that occurred during a Pap smear was a challenge for some participants. |  |  |
| 38.The long waiting time |  |  |
| 41.Inadequate health education about cervical cancer prevention and management |  |  |
| 43.Long waiting time |  |  |
| 44.Inadequate space |  |  |
| 45.Personnel problem |  |  |
| 46.Logistics |  |  |
| 51.Clinician Screening and Treatment Preparation |  |  |
| 52.Clinician Challenges and Barriers |  |  |
| 55.Not having all the equipment to perform the cervical smear |  |  |
| 56.Clinic procedures were inconsistent with some operating without appointments |  |  |
| 57.Long queues and waiting times |  |  |
| 58.Shortage of nurses |  |  |
| 59.Not fully explain the significance of an abnormal result or speak in a language that the woman could not understand |  |  |
| 74.High patient volume |  |  |
| 94.The centres currently available were far and few |  |  |
| 103.The lengthy line to take the test |  |  |
| 105.Lack of readiness for the screening |  |  |
| 8.Lack of continuum of care. | Lack of Continuous Support |  |
| 42.Lack of a proper follow-up mechanism |  |  |
| 54.Many participants never received their result or only obtained it months or years later |  |  |
